# Dynamic expression of leukocyte innate immune genes in whole blood from horses with lipopolysaccharide-induced acute systemic inflammation

**DOI:** 10.1186/s12917-015-0450-5

**Published:** 2015-06-16

**Authors:** Anne Mette L Vinther, Kerstin Skovgaard, Peter MH Heegaard, Pia H Andersen

**Affiliations:** Department of Large Animal Sciences, Faculty of Health and Medical Sciences, University of Copenhagen, Taastrup, Denmark; Innate Immunology Group, Section for Immunology and Vaccinology, National Veterinary Institute, Technical University of Denmark, Frederiksberg, Denmark; Department of Clinical Sciences, Faculty of Veterinary and Animal Science, Swedish University of Agricultural Sciences, Uppsala, Sweden

**Keywords:** Baseline expression variability, Cytokines, Endotoxemia, Equine, Gene expression dynamics, Innate immunity, Leukocytes, Lipopolysaccharide, Systemic inflammation, Time course study

## Abstract

**Background:**

In horses, insights into the innate immune processes in acute systemic inflammation are limited even though these processes may be highly important for future diagnostic and therapeutic advances in high-mortality disease conditions as the systemic inflammatory response syndrome (SIRS) and sepsis. Therefore, the aim of this study was to investigate the expression of 31 selected blood leukocyte immune genes in an equine model of acute systemic inflammation to identify significantly regulated genes and to describe their expression dynamics during a 24-h experimental period. Systemic inflammation was induced in 6 adult horses by the intravenous injection of 1 μg lipopolysaccharide (LPS) per kg btw. Sixteen blood samples were collected for each horse at predetermined intervals and analyzed by reverse transcription quantitative real-time PCR. Post-induction expression levels for each gene were compared with baseline levels.

**Results:**

Systemic inflammation was confirmed by the presence of clinical and hematological changes which were consistent with SIRS. The clinical response to LPS was transient and brief as all horses except one showed unaltered general demeanor after 24 h. Twenty-two leukocyte genes were significantly regulated at at least one time point during the experimental period. By close inspection of the temporal responses the dynamic changes in mRNA abundance revealed a very rapid onset of both pro- and anti-inflammatory mediators and a substantial variation in both expression magnitudes and duration of changes between genes. A majority of the 22 significantly regulated genes peaked within the first 8 h after induction, and an on-going, albeit tightly controlled, regulation was seen after 24 h despite approximate clinical recovery.

**Conclusions:**

This first broad study of gene expressions in blood leukocytes during equine acute LPS-induced systemic inflammation thoroughly characterized a highly regulated and dynamic innate immune response. These results provide new insights into the molecular mechanisms of equine systemic inflammation.

**Electronic supplementary material:**

The online version of this article (doi:10.1186/s12917-015-0450-5) contains supplementary material, which is available to authorized users.

## Background

High mortality rates in the equine clinic are related to the development and progression of severe systemic inflammatory conditions like the systemic inflammatory response syndrome (SIRS) and sepsis. This is seen in diseases as metritis, colitis, small intestinal strangulation, large intestinal volvulus, pleuropneumonia, and intrauterine/neonatal infections [1]. The clinical presentation in SIRS and sepsis is mainly caused by the immunological host response [2], which is orchestrated by highly dynamic and complex interactions of a vast number of cytokines, hormones, growth factors, and pattern recognition receptors derived from immunologically active cells, including leukocytes and endothelial cells [2, 3]. The outcome of an individual inflammatory response is therefore closely related to the specific immunological capacity of the host [4].

The clinical signs of systemic inflammation are nonspecific [1, 5], and the relation between clinical signs and the immunological response is not known. Consequently, an exact and timely status of an inflammatory condition is extremely difficult to obtain on the basis of clinical parameters, and insights into the early immunological disease processes may hold the key for future diagnostic and therapeutic advances in SIRS and sepsis.

In human medicine, decades of specific research in SIRS and sepsis have advanced the understanding of the underlying cellular processes in human systemic inflammation which, however, does not translate directly into the equine [6]. In foals, several studies have been conducted comparing innate gene expressions in blood leukocytes in septic patients versus non-septic [7–9] and healthy foals [10, 11]. In adult horses leukocyte immune genes have been correlated to type of disease and outcome in patients with gastrointestinal inflammation and strangulation [12] while a small number of studies have had a primary aim of investigating leukocyte innate immune gene expressions in experimental lipopolysaccharide- (LPS-) induced systemic inflammation [13–15]. These studies all encourage further investigations of the diagnostic potential of the immunological disease processes in equine systemic inflammation. However, also common for these studies is that a limited number of genes have been subjected to investigation, and that the highly dynamic nature of the inflammatory expression response has been less attended to. Controlled studies investigating the temporal responses of more complex gene interactions during equine systemic inflammation are therefore of interest.

The aim of this study was to investigate the expression of 33 selected blood leukocyte immune genes in an equine model of LPS-induced acute systemic inflammation to identify significantly regulated genes and to thoroughly describe the expression dynamics during the first 24 h of the disease course. The selected genes included proteins representing important innate functions such as amplification of the inflammatory reaction, pathogen associated molecular pattern recognition, cell adhesion, apoptosis, signal transduction, and oxidative burst. The overall aim was to expand our understanding of the innate immune processes involved in acute systemic inflammation in horses to advance equine clinical research in systemic inflammatory conditions as SIRS and sepsis.

## Methods

### Horses

Six healthy, adult (7.7 ± 5.4 years) Danish Warmblood or Danish Warmblood cross bred horses (2 geldings, 4 non-pregnant mares) with an initial body weight of 490 ± 39 kg and body condition score [16] of 4.5 ± 0.8 were included in the study. Before the experiment a complete clinical examination with standard blood hematological and biochemical analyses was performed. Only horses with unremarkable clinical and laboratory findings [17] were included in the study. Horses were housed in individual 3 x 4 m box stalls in a barn maintained at a temperature of 12 ± 1 °C. They were fed an equine commercial grain mixture twice daily and had access to water and hay ad libitum. All horses were dewormed with ivermectin (Maximec®) and vaccinated against equine influenza and tetanus (ProteqFlu-Te®) prior to the study.

### Experimental design

The horses were part of a larger randomized experimental cross over study involving LPS-induced endotoxemia and synovitis. Horses included in the present study received an intravenous injection of 1 μg LPS/kg bwt before or after an intra-articular administration of 3 μg LPS. Treatments were given on two separate occasions with a four week wash out period in between to eliminate possible effects of LPS tolerance. LPS derived from *Escherichia coli* strain 055:B5 (#L2880, Sigma-Aldrich Denmark) was diluted in isotonic saline to a total volume of 15 ml and administered over one minute through a jugular vein catheter. Blood samples for RNA extraction were collected in PAXgene Blood RNA Tubes (Qiagen/BD Company) before induction at times −120, −96, −24, and 0 h, and at post-induction hour (PIH) ½, 1, 1½, 2, 2½, 3, 3½, 4, 5, 6, 8, 10, 12, 16, 20, and 24. All 4 pre-induction samples were taken in the morning, and the sample at PIH 0 was taken within half an hour before LPS-injection. The first 5 ml blood were drawn in a separate syringe and discarded. According to the manufacturer’s instruction PAXgene blood tubes were gently inverted 8–10 times after sampling and kept at room temperature for 2 to 24 h before storage at −80 °C until mRNA extraction. All experimental procedures were approved by the Danish Animal Experiments Inspectorate (2011/561 − 1996) and carried out in agreement with the Danish Animal Testing Act.

### Evaluation of clinical and hematological signs

Examinations comprising general condition, rectal temperature (RT), heart rate (HR), respiratory rate (RR), borborygmus score, whole blood white blood cell count (WBC), and total neutrophil, lymphocyte, and monocyte counts were performed serially according to Table 1 to evaluate soundness of horses during the pre-induction period (PIH −120 to PIH 0) and to monitor post-induction disease progression and severity. Borborygmus was assessed on the ventral and dorsal abdomen on both sides and scored as ileus = 0, significantly decreased = 1, slightly decreased = 2, normal = 3. Thus, in total borborygmus score per horse for each time point ranged 0 (complete ileus) to 12 (normal borborygmus). Total and differential counts of white blood cells were measured with an automated cell counter (ADVIA 2120 hematology analyser, Siemens Healthcare Diagnostics Inc., Deerfield, Illinois, USA). At PIH 2, the following criteria were used to confirm SIRS: the presence of two or more of the following symptoms; RT < 36.7 or > 38.6 °C, HR > 50 beats/min, RR > 25 breaths/min, and WBC < 5.000 or > 14.500 cells/mm^3^ [1].

**Table 1:** Clinical and hematological evaluations during pre-induction and post-induction periods (mean ± SD)

	PIH −120	PIH −96	PIH −24	PIH 0	PIH 1	PIH 2	PIH 4	PIH 8	PIH 16	PIH 24	Ref
Rectal temperature (°C)	37.9 ± 0.3	37.5 ± 0.4	37.7 ± 0.2	37.8 ± 0.3	38.1 ± 0.5	38.9 ± 0.9^	39.9 ± 0.9^	38.4 ± 0.3	38.4 ± 0.2	38.1 ± 0.5	37.3 - 38.3
Heart rate (beats per minute)	38 ± 4	35 ± 5	37 ± 5	40 ± 8	52 ± 9	56 ± 8^	43 ± 8	50 ± 5	54 ± 9^	53 ± 13^	28 - 40
Respiratory rate (breaths per minute)	18 ± 11	14 ± 2	13 ± 4	16 ± 4	–	18 ± 8	17 ± 5	28 ± 22	12 ± 5	15 ± 8	10 -14
Borborygmus score^a^	11.3 ± 0.8	11.5 ± 0.8	11.7 ± 0.8	11.7 ± 0.5	3.8 ± 2.9^	3.8 ± 3.3^	6.5 ± 2.5^	9.5 ± 2.9	9.8 ± 3.3	9.8 ± 4.4	
WBC (10^9 cells/L)	8.2 ± 1.7	7.1 ± 1.4	8.0 ± 2.0	7.5 ± 1.7	2.0 ± 0.9^	1.7 ± 1.0^	2.7 ± 1.6^	9.5 ± 3.2	12.9 ± 2.8^	14.1 ± 2.7^	6 - 12
Total neutrophil count (10^9 cells/L)	4.5 ± 0.7	3.5 ± 0.5	4.0 ± 1.1	4.0 ± 0.9	0.2 ± 0.1^	0.3 ± 0.3^	1.6 ± 1.3	8.4 ± 2.9^	10.9 ± 2.2^	11.6 ± 2.5^	3 - 6
Total lymphocyte count (10^9 cells/L)	3.0 ± 1.0	2.9 ± 1.1	3.1 ± 1.1	2.8 ± 1.1	1.7 ± 0.8	1.3 ± 0.7^	1.0 ± 0.4^	0.7 ± 0.4^	1.5 ± 1.0^	2.1 ± 1.3	1.5 - 5
Total monocyte count (10^9 cells/L)	0.4 ± 0.2	0.4 ± 0.1	0.4 ± 0.2	0.4 ± 0.2	0.1 ± 0.03^	0.04 ± 0.03^	0.04 ± 0.03^	0.1 ± 0.04^	0.2 ± 0.1^	0.2 ± 0.1^	0 - 0.6

Pre-induction period: PIH −120 – PIH 0. Post-induction period: PIH 1 – PIH 24. ^a^Borborygmus was assessed on the ventral and dorsal abdomen on both sides and scored as ileus = 0, significantly decreased = 1, slightly decreased = 2, normal = 3. In total, borborygmus score per horse for each time point ranged 0 (complete ileus) to 12 (normal borborygmus). ^ denotes statistical significant difference (p < 0.0063) of post-injection levels compared with baseline levels at PIH 0. Reference values (ref) are according to Aiello [17]

### Target genes

Relative gene expression in blood leukocytes were established by quantification of specific mRNA for the following genes: *IL1B*, *IL1RN*, *IL2*, *IL4*, *IL6*, *IL6ST*, *IL8*, *IL10*, *IL15*, *IL17*, *IL18*, *TNF*, *TLR4*, *TLR9*, *SELL*, *ITGAM*, *ITGAX*, *TGFB*, *HMGB1*, *MIF*, *CD14*, *NKAP*, *MAPK14*, *FAS*, *BID*, *CASP3*, *BCL2L1*, *MPO*, *MMP8*, *TIMP1*, *CCL5*, *SOD2*, *GSF2* (gene names and functional classes are listed in Additional file 1).

### Total RNA extraction and quality analysis

Total cellular RNA was extracted from PAXgene blood samples using PAXgene Blood miRNA Kits (Qiagen/BD Company) according to the manufacturer’s instructions. All RNA samples were treated with RNase-Free DNase sets (Qiagen/BD Company). Concentration and purity of total extracted RNA was determined by spectrophotometric analyses (NanoDrop ND-1000 spectrophotometer, Saveen and Werner AB, Limhamn, Sweden). The concentration was measured at optical density (OD)_260_, while assessment of purity was based on OD_260/280_ and OD_260/230_ ratios. Samples containing less than 20 ng RNA/μL (9 % of samples) were evaporated (37 °C) to increase RNA concentrations using a SpeedVac Concentrator (SPD111V, Thermo Scientific, Slangerup, Denmark). RNA integrity was estimated via capillary electrophoresis in an Agilent 2100 Bioanalyzer (Agilent Technologies, Naerum, Denmark) using RNA 6000 Nano Kits (Agilent Technologies). Each total RNA sample was assigned an RNA Integrity Number (RIN) from 1–10, with 10 being non-degraded RNA [18]. Mean RIN value ± SD of all samples was 8.7 ± 0.9.

### cDNA synthesis

Samples containing extracted RNA were DNase treated to eliminate genomic DNA and converted into first-strand cDNA by reverse transcription using a Tprofessional TRIO 3x48 (Fisher Scientific) and QuantiTect Reverse Transcription Kits (Qiagen/BD Company) according to the manufacturer’s instructions. In brief, 300 ng of total RNA was DNase treated by 2 min incubation at 42 °C. Reverse transcriptase enzyme and a mix of random primers and dNTPs (1:4) were added, and samples incubated for 15 min at 42 °C. Finally, enzymes were denatured for 3 min at 95 °C and cDNA cooled down to 4 °C. Two separate cDNA replicates were performed for each sample and a non-reverse transcriptase control included. The cDNA samples were diluted (1:7.7) in low TE-buffer (VWR-Bie & Berntsen, Herlev, Denmark) and stored at −20 °C until pre-amplification procedures.

### Specific primer design

Gene specific primer pairs were designed using Primer3 (http://frodo.wi.mit.edu/primer3). Nucleic acid sequences were obtained from a free online genome database (http://www.ensembl.org/Equus_caballus/Info/Index). BLAST (http://blast.ncbi.nlm.nih.gov/Blast.cgi) searches were performed to ensure the absence of intraspecies polymorphisms at the primer site. When possible, primers were designed to span an intron to prevent possible amplification of contaminating genomic DNA. Primers were synthesized at TAG Copenhagen (Copenhagen, Denmark) or Sigma-Aldrich (Denmark). Transcript IDs, primer sequences, amplicon lengths, reaction efficiencies, and correlation coefficients are shown in Additional file 1. Primer amplification efficiencies, correlations and dynamic ranges were acquired from standard curves constructed from three separate dilution series of pooled pre-amplificated, exonuclease treated cDNA with the following dilutions: 1:3, 1:9, 1:27, 1:81, 1:243, and 1:729. The pool consisted of cDNA from every 4th sample representing different sample times, animals, treatments, and cDNA-replicates. After completed qPCR many of the samples showed a very low expression level of IL-2 and IL-6. To cover the dynamic range of IL-2 and IL-6 an additional standard curve was made from a dilution series of a pool of cDNA samples showing high expression levels of IL-2 and IL-6 to calculate primer amplification efficiencies and correlations for these specific genes. Efficiencies between 0.91 and 1.12 and correlations above 0.97 were accepted.

### Pre-amplification and exonuclease treatment

A 200 nM primer pair mix in low TE-buffer was prepared combining equal amounts of all primers used in the study (Additional file 1). Five μL of TaqMan PreAmp Master Mix (Applied Biosystems), 2.5 μL of the primer pair mix, and 2.5 μL of cDNA were mixed and incubated at 95 °C for 10 min followed by 16 cycles of 95 °C for 15 s and 60 °C for 4 min. To remove excess nucleotides pre-amplified cDNA was incubated with 4 μL of 4U/μL exonuclease for 30 min at 37 °C, followed by 15 min at 80 °C. An aliquot of the cDNA was saved for preparation of a dilution series, and finally cDNA was diluted 1:7 in low TE-buffer before quantitative real-time PCR (qPCR).

### qPCR

Quantitative PCR was performed in a 48.48 Dynamic Array Integrated Fluidic Circuits (IFC) controller (Fluidigm, CA, USA), which combines 48 samples with 48 primer sets in 2304 separate, simultaneous reactions as previously described in Skovgaard et al. [19]. In brief, for each of the 48 cDNA sample lanes on the Dynamic Array chip, a ‘sample mix’ consisting of 1.5 μL of specific pre-amplified exonuclease-treated cDNA, 3 μL of TaqMan Gene Expression Master Mix (Applied Biosystems), 0.3 μL of 20X DNA Binding Dye Sample Loading Reagent (Fluidigm), 0.3 μL 20X EvaGreen (Biotium, VWR-Bie & Berntsen), and 0.9 μL low TE-buffer was prepared. For each of the 48 primer set lanes on the chip, a ‘primer mix’ consisting of 2.3 μL of a specific 20 μM forward/reverse primer (see Additional file 1), 2.5 μL of 2X Assay Loading Reagent (Fluidigm), and 0.25 μL low TE-buffer was prepared. After priming the 48.48 Dynamic Array chip in an IFC controller (Fluidigm), it was loaded with cDNA sample mixes and primer mixes and again placed in the IFC controller to combine each of the 48 samples with each of the 48 primers. The chip was subsequently placed in a BioMark real-time PCR Reader (Fluidigm), where qPCR was performed under the following conditions: 2 min at 50 °C, 10 min at 95 °C (heat activation) followed by 35 cycles of 15 s at 95 °C (denaturation) and 1 min at 60 °C (annealing/elongation). After the last cycle melting curves were generated by heating from 60 °C to 95 °C (increasing 1 °C/3 s) to confirm a single PCR product. Each chip included a non-template control (NTC), a non-reverse transcription control and three interplate calibrators. Expression data (Cq values) and melting curves were acquired using Fluidigm Real-Time PCR Analysis software 3.0.2 (Fluidigm). NTCs and melting curves were used to monitor for non-specific amplification or sample contaminations. Non-reverse transcriptase controls were used to assess potential DNA contamination. For both control samples, a minimum of 5 Cq-values between potential signals and sample signal were required and only genes with a single melting peak were accepted for further data analyses.

### Pre-processing of data

Expression data were exported to GenEx5 (MultiD, Göteborg, Sweden) for data pre-processing. Data was interplate calibrated and corrected for primer efficiencies for each primer assay individually. Normalization was done to the reference genes *ACTB*, *TBP*, *DIMT1*, *SDHA*, *HPRT1*, *and B2M*. These genes were identified as the most stably expressed reference genes out of 7 candidates using NormFinder [20] and GeNorm [21]. cDNA technical replicates were averaged, and for a specific primer set a maximum of 15 % samples with a ΔCq above ±1.2 for the two cDNA replicates was accepted. For a specific sample a maximum of 20 % primer sets with a ΔCq above ±1.2 was accepted. Finally, relative expression levels were established. For each gene the expression level was set to 1 for the sample with the lowest level of expression. Expression levels for the specific gene in all other samples, irrespective of horse and time for sampling, were then calculated relative to this sample during data transformation from Cq (log2) to relative quantities (RQ) (relative fold change, linear scale).

### Descriptive and statistical analyses

Statistical analyses of post-injection levels of clinical and hematological parameters compared with baseline levels at PIH 0 were performed using one-way repeated measures ANOVA and Dunnett’s post hoc test (SigmaPlot v. 12.5.). Distribution of data was evaluated by the Shapiro-Wilk normality test and data was log2-transformed to improve the model fits. Using the Bonferroni correction to account for multiple testing, statistical significance was defined as P < 0.0063 (significance level 0.05/8 parameters) for all analyses.

Relative quantities (raw data) were depicted graphically using SigmaPlot v. 12.5. Baseline expression levels for each gene were tested for variability in the 4 pre-induction samples. Post-induction expression levels for each gene were compared with baseline levels at PIH 0 and significant regulation defined as statistically significant differential expression with a change of at least 2.5 fold. For both baseline samples and post-induction samples statistical differential expression was determined using a linear mixed model with “time” as fixed effect, “horse” as random effect (random intercept), and Dunnett’s post hoc test (R v. 3.1.1.) on log2-transformed data. Distribution of data was evaluated by Shapiro-Wilk normality tests and Q-Q plots of the residuals and found to be approximately log2-normally distributed. Using the Bonferroni correction to account for multiple testing, statistical significance was defined as P < 0.0016 (significance level 0.05/31 genes) for all analyses.

## Results

### Clinical and hematological responses

All horses showed characteristic signs of endotoxemia. A summary of clinical and hematological recordings and statistical results are listed in Table 1. During the pre-induction period all horses were bright, alert, and responsive, and considered healthy on clinical and hematological examinations. Within the first hour post-induction all horses started to express signs of general discomfort including anorexia and mild to moderate colic (watching flanks/kicking/lying down/rolling). At PIH 1 and PIH 2 fever, tachycardia, severely decreased borborygmus, and profound leukopenia were recorded, and all horses could be classified as having SIRS. At PIH 4 and afterwards the most prominent alterations in general demeanor were decreased appetite, depression (with/without recumbency), and mild colicky behavior. Fever peaked at PIH 4 and WBC and borborygmus started to increase. At PIH 24 all horses except one were bright, alert, and responsive, despite ongoing leukocytosis and increased HR. All horses recovered without the need for treatment. As seen in Table 1 changes in post-induction WBC primarily reflected neutropenia (PIH 1 – PIH 4) followed by neutrophilia (PIH 8 – PIH 24). Decreased total monocyte and to a lesser degree lymphocyte counts were also seen during the full post-induction experimental period including a steep decline in monocyte count at PIH 1.

### Gene expressions

In the pre-processing of data, three blood samples (2.9 %) and 4 single expression measurements for a specific gene (0.1 %) were excluded due to sampling error or high ΔCq values between technical replicates. The genes *GSF2* and *MPO* were not subjected to further analyses due to high sensitivity to genomic DNA contamination, indications of splice variants, or low primer efficiency. Thus, a total of 31 genes were evaluated for differential expression after induction of systemic inflammation.

For each of the 31 genes except *TIMP1* (p-value 0.0013) mean expression levels in 4 consecutive baseline samples were statistically equivalent. Post-induction mean expression levels for each gene are depicted in relative quantities as a function of time in Additional file 2. Table 2 sums up the overall analyses for each gene of the post-induction expression levels compared with pre-induction levels at PIH 0. All genes except *IL2* showed a statistically significant differential expression at minimum one time point after LPS-injection (Table 2A). Out of these the following 22 genes had expression levels ≥ 2.5 fold changes compared with baseline levels: *IL1B*, *IL1RN*, *IL6*, *IL6ST*, *IL8*, *IL10*, *IL15*, *IL17*, *IL18*, *TNF*, *TLR4*, *SELL*, *ITGAM*, *ITGAX*, *CD14*, *MAPK14*, *CASP3*, *BCL2L1*, *MMP8*, *TIMP1*, *CCL5*, and *SOD2* (Table 2B). Table 2C, D, and E list up-regulated and down-regulated genes and genes with alternated up- and down-regulations, respectively.

**Table 2:** Evaluation of the overall post-induction expression levels compared with pre-induction levels at PIH 0

All genes	2A: Genes with statistically significant differential expression	2B: Genes with statistically significant differential expression ≥ 2.5 fold	2C: Genes with statistically significant up-regulation ≥ 2.5 fold	2D: Genes with statistically significant down-regulation ≥ 2.5 fold	2E: Genes with statistically significant mixed up- and down-regulation ≥ 2.5 fold
*IL1B*	*IL1B*	*IL1B*	*IL1B*		
*IL1RN*	*IL1RN*	*IL1RN*	*IL1RN*		
*IL2*					
*IL4*	*IL4*				
*IL6*	*IL6*	*IL6*	*IL6*		
*IL6ST*	*IL6ST*	*IL6ST*	*IL6ST*		
*IL8*	*IL8*	*IL8*	*IL8*		
*IL10*	*IL10*	*IL10*	*IL10*		
*IL15*	*IL15*	*IL15*		*IL15*	
*IL17*	*IL17*	*IL17*			*IL17*
*IL18*	*IL18*	*IL18*			*IL18*
*TNF*	*TNF*	*TNF*	*TNF*		
*TLR4*	*TLR4*	*TLR4*	*TLR4*		
*TLR9*	*TLR9*				
*SELL*	*SELL*	*SELL*			*SELL*
*ITGAM*	*ITGAM*	*ITGAM*	*ITGAM*		
*ITGAX*	*ITGAX*	*ITGAX*	*ITGAX*		
*TGFB1*	*TGFB1*				
*HMGB1*	*HMGB1*				
*MIF*	*MIF*				
*CD14*	*CD14*	*CD14*			*CD14*
*NKAP*	*NKAP*				
*MAPK14*	*MAPK14*	*MAPK14*			*MAPK14*
*FAS*	*FAS*				
*BID*	*BID*				
*CASP3*	*CASP3*	*CASP3*			*CASP3*
*BCL2L1*	*BCL2L1*	*BCL2L1*	*BCL2L1*		
*MMP8*	*MMP8*	*MMP8*	*MMP8*		
*TIMP1*	*TIMP1*	*TIMP1*	*TIMP1*		
*CCL5*	*CCL5*	*CCL5*		*CCL5*	
*SOD2*	*SOD2*	*SOD2*			*SOD2*

2A: genes with a statistically significant differential expression at minimum one time point after LPS-induction. 2B: genes with statistically significant differential expression of at least 2.5 fold. 2C, 2D, and 2E: genes with statistically significant up-regulation, down-regulation, and mixed up- and down-regulation, respectively, of at least 2.5 fold. All genes listed in 2A – 2E had p-values below 0.0001

**Fig. 1:**
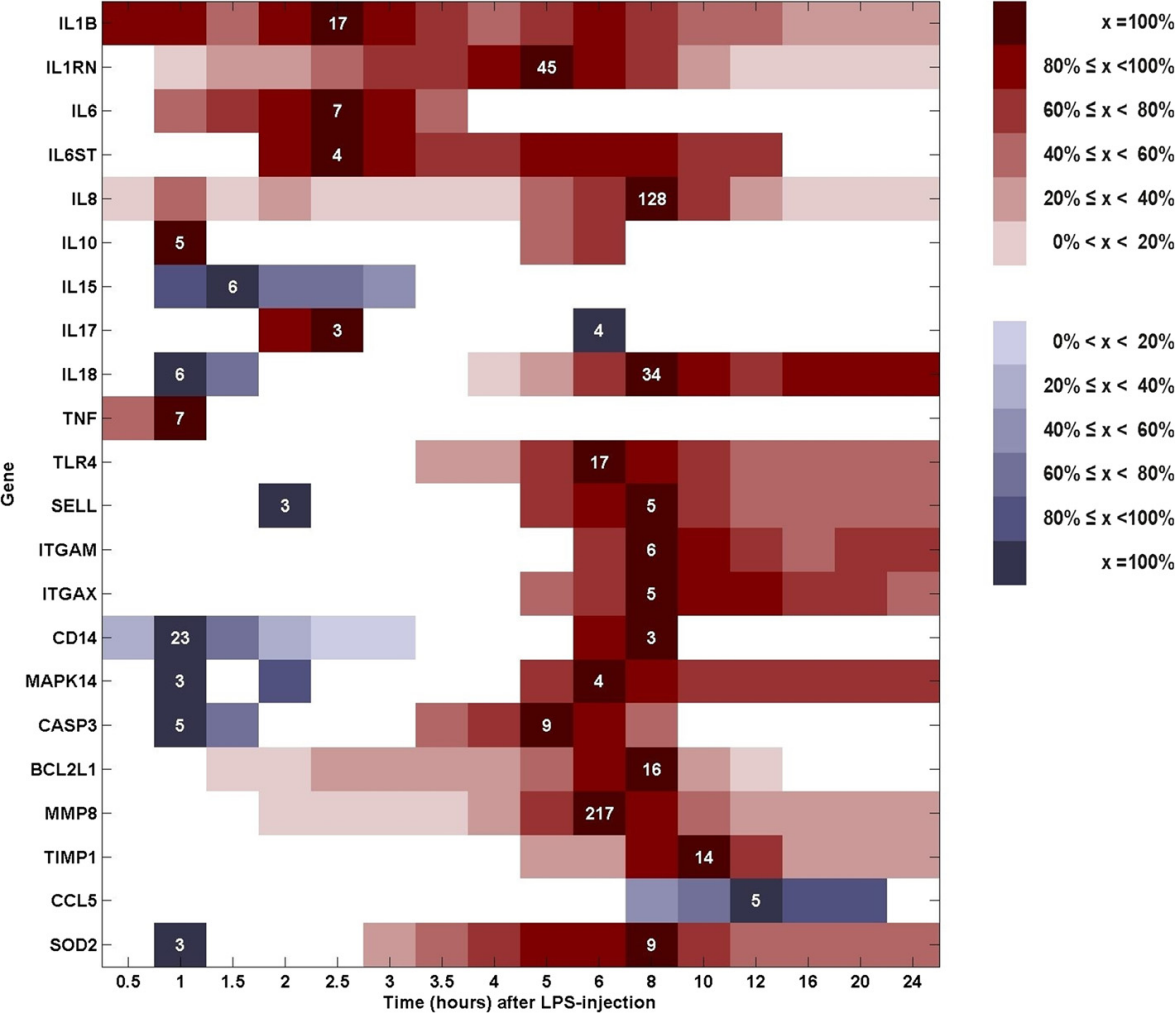
Expression dynamics during the 24-h experimental period. Statistically significant differential expressions of at least 2.5 fold compared with baseline levels at PIH 0 are depicted as a function of time (rounded to nearest integer). Up-regulations are illustrated in red shades and down-regulations in blue shades. For each gene, peak up- and down-regulations in fold change are stated in white letters. All other expression levels are depicted in red or blue shades, respectively, in percent fold change relative to peak fold change. Time points without a statistically significant differential expressions of at least 2.5 fold compared with PIH 0 are left white

Statistically significant differential expressions of at least 2.5 fold are depicted as a function of time in Fig. 1 to show the expression dynamics during the full 24-h sampling period. Up-regulations are illustrated in red colors and down-regulations in blue colors. For each gene, peak up- and down-regulations in fold change compared with PIH 0 are stated in white numbers. All other expression levels are depicted in red and blue shades for up- and down-regulated genes, respectively, corresponding to percent fold change relative to peak fold change. Time points without a statistically significant differential expressions of at least 2.5 fold compared with PIH 0 are left white. Significant regulation of mRNA expression levels were seen in the whole 24 h sampling period. The first genes to be differentially expressed were the pro-inflammatory genes *IL1B*, *IL8*, *TNF* (up-regulated), and *CD14* (down-regulated). *CCL5* was the most slow-reacting gene with a transcription onset at PIH 8. Most up- and down-regulations peaked within the first 8 h with a high incidence of up-regulated peaks between PIH 5 and PIH 8. After PIH 8 only *TIMP1* and *CCL5* showed its peak expression. Magnitudes of mean peak expression varied within 217 fold up-regulation (*MMP8*) and 23 fold down-regulation (*CD14*). A slight majority of the genes were still differentially expressed at PIH 24 although most of them were only weakly to moderately expressed at this time point.

## Discussion

Innate leukocyte immune gene expressions have only been scarcely studied in in vivo equine systemic inflammation. Here, we investigated the expression of 31 selected genes in a serial measurements design to put special emphasis on the temporal changes of expression during the disease course of LPS-induced acute systemic inflammation. Based on number of genes and samples, the dynamics of equine gene expressions have not been characterized to this extension before.

Intravenous injection of LPS is a well described model to induce systemic inflammation in horses and leads to a consistent response including i.a. fever, tachycardia, tachypnea, and leukopenia followed by leukocytosis [22–26]. All horses in this study expressed these classic signs of LPS-induced systemic inflammation (Table 1) and could be classified as having SIRS. They all showed the most severe clinical signs in the first 2 to 4 h, after which their clinical status gradually improved. The disease was self-limiting with no need for therapeutic intervention, and at the end of the 24-h post-induction sampling period all horses except for one showed an unaltered general demeanor. A similar clinical course for horses subjected to intravenous LPS was described in Jacobs et al. [26]. The brief clinical response reflects that LPS was given as a single bolus as it only exerts its direct effects on the body for a short period of time due to rapid clearance from circulation [27]. Horses and humans show comparable responses to LPS, and a similar course of a well-defined physiological response of approximately 24 h duration is reported from healthy humans subjected to LPS-induced systemic inflammation [28–30].

All investigated genes except *IL2* showed a statistically significant differential expression compared with pre-induction levels at PIH 0 at minimum one time point after LPS-induction. When a criterion of a differential expression of at least 2.5 fold for assumed biological relevance was applied, *IL4*, *TLR9*, *TGFB*, *HMGB1*, *MIF*, *NKAP*, *FAS*, and *BID* were filtered out. Thus, the 22 genes listed in Table 2B were found to be significantly regulated in this case of equine LPS-induced acute systemic inflammation and consequently regarded relevant for the orchestration of the inflammatory response. A biological relevance of genes with a statistically significant regulation below 2.5 fold can, however, not be excluded.

Magnitudes of the observed fold changes differed substantially between genes with *MMP8* showing the greatest up-regulation with a peak of 217 fold change compared with baseline levels. This is in line with human studies of septic shock in children where *MMP8* consistently was the highest expressed gene [31]. The most down-regulated gene, *CD14*, also showed a prominent regulation with a 23 fold change compared with pre-induction levels. However, the biological importance of expression magnitudes in the systemic inflammatory response is unclear. In general, there is not a strong correlation between the cellular levels of specific proteins and the abundances of their corresponding mRNA [32–34], and the biological effects of a highly differentially expressed gene might not be different from a gene with a lower peak expression value. As an example, *IL10* in this study is only moderately differentially expressed compared with many of the other genes despite that it is known to be a key moderator of systemic inflammation. Thus, it might be more explanatory to the orchestration of systemic inflammation to explore the expression dynamics over time, and in the present study numerous repeated samplings after a single bolus of LPS enabled the investigation of the dynamic gene expressions during a full disease course of acute systemic inflammation from clinical onset to approximate recovery.

The very diverse and dynamic expression patterns depicted in Fig. 1 confirm the complexity of the inflammatory response. Many of the genes showed alternations between up- and down-regulations, and while some remained differentially expressed during the full disease course, others did not and may thus only add to the inflammatory response at certain disease stages. The early leukocyte response to LPS had a rapid onset as several of the pro-inflammatory genes showed differential expression at PIH ½. The response seemed to be very delicately regulated as the time span between down- and up-regulations was as short as 2 h (*CASP3*, PIH 1.5 – 3.5). Just like the clinical response, the gene expressions reflected the moderate, single-hit challenge. Almost all genes showed peak regulation within the first third (PIH 8) of the full experimental period, and at PIH 24 – where all horses except one showed unaltered general demeanors – many of the genes had returned to baseline levels or to levels of moderate differential expression. This is in accordance with expression studies of human LPS-induced systemic inflammation of similar duration [28, 29, 32]. As an example, Calvano et al. [28] reported in a microarray study that a number of genes were induced at PIH 2, that other genes peaked at PIH 4–9, and that they all had returned to baseline levels by PIH 24.

Considering the clinical well-being of the horses at PIH 24 it was somewhat surprising that many of the pro-inflammatory genes still showed differential expression at this time point. However, as the half-life of mRNA for cytokines and transcriptional activators are short [35] and as transcription of genes is very rapidly regulated, the measured expression levels indicate a true on-going inflammatory reaction. As LPS can only exert its direct effects on leukocytes during the very short endotoxemic period preceding clearance [27], it supports that a pathogen-associated molecular pattern (PAMP) initiated immune reaction is self-sustained for a period of time by released cytokines and endogenous danger-associated molecular patterns (DAMPs). Due to the termination of the study after 24 h, this study does not elucidate whether altered expression patterns are present in horses after resolution of all clinical symptoms. In a study performed by Fossum et al. [14] leukocyte *TLR4* and *LY96* (alias *MD-2)* expression levels were elevated 48 h after the beginning of a 6-h continuous intravenous LPS-infusion. However, no information on the clinical status of the horses after 48 h was included.

Generally, genes involved in host responses may be divided into fast reacting genes and genes with a delayed response as it is done in Calvano et al. [28]. In the present study, some of the “classic” cytokine genes like *IL1B*, *IL6*, *IL8*, *IL10*, and *TNF* showed a very rapid transcription onset with, except for *IL8*, a peak up-regulation coinciding with early clinical symptoms such as tachycardia, severely decreased borborygmus, and profound leukopenia. The pro-inflammatory genes *IL1B*, *IL6*, *IL8*, and *TNF* have shown a similar rapid response to LPS in other equine LPS-studies [13, 26, 36] as well as in human studies [32] while *IL10* showed a more delayed response [32, 36]. Other genes with a rapid transcription onset (within 3 h) are *IL1RN*, *IL6ST*, *IL17*, *BCL2L1*, *MMP8*, and *SOD2*.

Anti-inflammatory mediators (*IL10* and *IL1RN*) and pro-inflammatory mediators (*IL1B*, *IL6*, *IL8*, and *TNF*) both showed transcription onsets within the first hour of LPS-injection indicating that these two counteracting systems are initiated in temporal proximity. However, anti-inflammatory genes seemed to have a slightly delayed transcription onset and/or peak expression compared with the corresponding pro-inflammatory genes (*IL1B/IL1RN*, *TNF/IL10*). This is biologically plausible as an initial pro-inflammatory response to PAMPs must be tightly controlled after onset to avoid excessive amplification of these mediators. Damage to host tissues due to excessive, uncontrolled pro-inflammation is regarded one of the key factors in the pathogenesis of severe sepsis and septic shock [2, 37]. In this regard it is interesting that *MMP8*, which is involved in degradation of extracellular matrix, is differentially expressed for 2.5 h before up-regulation of the general metalloproteinase inhibitor, *TIMP1* [38]. This might, however, suggest the existence of more specific anti *MMP8* genes not tested in this panel. The anti-inflammatory *IL1RN*, *TIMP1*, and *SOD2* were all up-regulated in the late part of the experimental period supporting that a tight orchestration is necessary for restoration of immunological homeostasis.

Assessed by expression peaks apoptosis is counteracted by anti-apoptosis demonstrated by the genes *CASP3* and *BCL2L1*, respectively. This may reflects a tight regulation of apoptosis and anti-apoptosis pathways, and it is known that members of the TNF superfamily can activate both simultaneously [39]. The other apoptosis-related genes investigated, *FAS* and *BID*, were not differentially expressed above 2.5 fold in this study. Whether apoptosis primarily has protective or harmful consequences during systemic inflammation is still unknown [3] but *CASP3*, *BCL2L1*, *FAS*, and *BID* are all reported to be differentially expressed in human patients suffering from different stages of sepsis [40–42].

Down-regulation of *IL15*, *IL18*, *SELL*, *CD14*, *MAPK14*, *CASP3*, and *SOD2* was measured during the first hours after LPS-injection. This was somewhat unexpected as at least some of these mediators are known to be crucial for initiation of the inflammatory response to bacterial PAMPs as LPS. As an example, CD14 is part of the pattern recognition complex comprising lipopolysaccharide binding protein, CD14, TLR-4, and lymphocyte antigen 96 which contributes essentially to the initiation of the whole downstream inflammatory cascade in the presence of LPS [2]. However, the early down-regulations seen in this study do not necessarily reflect intrinsic differences in mRNA expression. Instead they may reflect shifts in the composition of circulating leukocyte subsets. Early leukopenia with relative lymphocytosis followed by neutrophilic leukocytosis was observed in the horses as seen in Table 1. Both leukocyte shifts are well-known phenomena in both LPS-induced and naturally occurring acute systemic inflammation [1] and are caused by neutrophil and monocyte margination/extravasation and release of neutrophil reserves from the bone marrow, respectively [43, 44]. As seen in Table 1 neutrophils and monocytes were very few in numbers in blood samples drawn in the first hours after the inflammatory onset. This may well explain why *CD14*, which is almost exclusively expressed by these two types of white blood cells, appears as down-regulated in the early inflammatory phase. However, the pro-inflammatory cytokines *IL1*, *IL8*, and *TNF* are mainly synthesized by the exact same cells expressing *CD14*. The observation of an early down-regulation of the expression of *CD14* but not of the pro-inflammatory gene expression would therefore suggest a dramatic increase in pro-inflammatory gene expression – but not in *CD14* expression – more than compensating for the decrease in circulating numbers of these cells. *TLR4* and the genes encoding adhesion molecules are also known to be involved in the initiation of the inflammatory response [2, 43], and the decrease in relative monocyte and neutrophil proportions in circulation blood may likewise explain why no up-regulation of these particular genes is observed in the early inflammatory response. Shifts in the circulating leukocyte composition might further account for some of the massive up-regulations seen across genes at PIH 5 – PIH 8 as especially neutrophils were getting abundant in circulating blood. The general abundance in transcribed mRNA at PIH 5 – PIH 8 can, however, not be entirely attributed to increases in total neutrophil and monocyte numbers as these do not peak until PIH 24 (Table 1).

Our results suggest that measurements of whole blood in vivo leukocyte gene expressions are dependent upon the composition of the various types of white blood cells extracted at a given time point during the disease course. This is supported by both equine [8] and human [45, 46] studies and is biologically plausible as various cell types have different capacities for expressing specific genes. As a result, caution is needed in extrapolating results from such studies directly to the pathogenesis of the systemic inflammatory response to endotoxin. In equine patients with naturally occurring systemic inflammation, leukopenia with profound neutropenia is however so consistent and well-documented that it is regarded pathognomonic for the acute stage of disease [1]. Gene expression analyses on horses with for example LPS-induced systemic inflammation may therefore very well be relevant for the exploration of molecular biomarker candidates in patients suffering from SIRS and sepsis.

Investigation of the immunological processes in systemic inflammation is a major research area in human medicine as it is believed to hold great promise for future diagnostic and therapeutic advancements in SIRS, sepsis, severe sepsis, and septic shock [47]. Many studies have identified specific differentially expressed genes in these disease conditions, either compared with healthy controls or with patients suffering from a different subgroup of systemic inflammation [48]. It has, however, proven extremely difficult to employ individual genes as biomarkers in clinical practice [48], and recent studies have turned towards a multi-biomarker approach to better encompass the complexity of systemic inflammation [49–51]. Relatively few leukocyte immune genes have until now been investigated in the equine, and the results presented here may contribute to future definition of a multi-biomarker candidate for early equine systemic inflammation. However, the present study investigated the innate immune response to a moderate, single-hit insult by LPS. A more biologically relevant experimental model might have included both Gram-negative and Gram-positive PAMPs given repeatedly or as continuous infusion. Naturally occurring sepsis might be initiated and maintained by repeated hits of a combination of PAMPs and endogenous DAMPs, as is thought to be the case in severely ill horses with ischemic intestinal segments and transmural migration of gut bacteria to circulating blood. However, the value of LPS studies is supported by DeClue et al. [52], who only reported of minor differential effects in the equine leukocyte cytokine response to combinations of in vitro LPS and Gram-positive PAMPs (lipoteichoic acid and peptidoglycan) compared with each of these motifs alone. In addition, important considerations to take on from this study to future studies are to relate expression levels to the concurrent composition of white blood cells, and to investigate expression dynamics over time as regulation of gene expressions is orchestrated in such a highly dynamic manner.

## Conclusions

This study investigated the expression of 33 selected blood leukocyte immune genes in a model of LPS-induced short-term systemic inflammation in horses. It was shown that 22 leukocyte genes encoding proteins involved in i.a. inflammatory reaction amplification, pathogen associated molecular pattern recognition, cell adhesion, apoptosis, signal transduction, and oxidative burst were significantly regulated at at least one time point during the 24-h experimental period which covered disease onset to approximate clinical recovery. By close inspection of the temporal responses, changes in mRNA abundance revealed a highly regulated and dynamic nature of this response. This first broad study of blood leukocyte gene expression in equine acute LPS-induced systemic inflammation provides new insights into the molecular mechanisms of naturally occurring equine systemic inflammation and may contribute to future biomarker development in SIRS and sepsis.

## Additional files

Additional file 1:
**Primers and reaction conditions.** PCR efficiencies and correlation coefficients for genes marked with (*) are calculated based on a specific high-responder standard curve made from dilution series of a pool of cDNA samples showing high expression levels of the implicated genes. Genes marked with (#) were excluded from the study due to high sensitivity to genomic DNA contamination, indications of splice variants, or low primer efficiency. r^2^ = correlation coefficient. Additional file 2:
**Post-induction mean expression levels for each gene.** Log2-transformed relative gene expressions (mean ± SD) depicted as a function of time in hours after LPS-injection. Expression levels are set relative to the lowest expression level of the gene in question. Time point 0 indicates baseline levels immediately before LPS-injection. Time after injection is depicted non-equidistantly.

## Abbreviations

DAMP: Danger associated molecular patterns
dNTP: Deoxyribonucleotide triphosphate
HR: Heart rate
LPS: Lipopolysaccharide
LY96: Lymphocyte antigen 96
mRNA: Messenger ribonucleic acid
MD-2: Myeloid differentiation protein-2
PAMP: Pathogen associated molecular patterns
PIH: Post-induction hour
RIN: RNA integrity number
RR: respiratory rate
RT: rectal temperature
RT-qPCR: Reverse transcription quantitative real-time polymerase chain reaction
RQ: Relative quantities
SIRS: Systemic inflammatory response syndrome
TE: Tris-ethylenediaminetetraacetic acid
WBC: White blood cell count

## References

[1] Mackay RJ. Endotoxemia. In P. SB editor, Large Animal Internal Medicine*.* 4. edn. St. Louis, Missouri: Mosby Elsevier; 2009: 711–23.

[2] Cohen J (2002). The immunopathogenesis of sepsis. Nature.

[3] van der Poll T, Opal SM (2008). Host-pathogen interactions in sepsis. Lancet Infect Dis.

[4] Gustot T (2011). Multiple organ failure in sepsis: prognosis and role of systemic inflammatory response. Curr Opin Crit Care.

[5] Bone RC, Balk RA, Cerra FB, Dellinger RP, Fein AM, Knaus WA (1992). Definitions for sepsis and organ failure and guidelines for the use of innovative therapies in sepsis. The ACCP/SCCM Consensus Conference Committee. American College of Chest Physicians/Society of Critical Care Medicine. Chest.

[6] Werners AH, Bull S, Fink-Gremmels J (2005). Endotoxaemia: a review with implications for the horse. Equine Vet J.

[7] Pusterla N, Magdesian KG, Mapes S, Leutenegger CM (2006). Expression of molecular markers in blood of neonatal foals with sepsis. Am J Vet Res.

[8] Castagnetti C, Mariella J, Pirrone A, Cinotti S, Mari G, Peli A (2012). Expression of interleukin-1beta, interleukin-8, and interferon-gamma in blood samples obtained from healthy and sick neonatal foals. Am J Vet Res.

[9] Gold JR, Cohen ND, Welsh TH (2012). Association of adrenocorticotrophin and cortisol concentrations with peripheral blood leukocyte cytokine gene expression in septic and nonseptic neonatal foals. J Vet Intern Med.

[10] Gold JR, Perkins GA, Erb HN, Ainsworth DM (2007). Cytokine profiles of peripheral blood mononuclear cells isolated from septic and healthy neonatal foals. J Vet Intern Med.

[11] Burton AB, Wagner B, Erb HN, Ainsworth DM (2009). Serum interleukin-6 (IL-6) and IL-10 concentrations in normal and septic neonatal foals. Vet Immunol Immunopathol.

[12] Lopes MA, Salter CE, Vandenplas ML, Berghaus R, Hurley DJ, Moore JN (2010). Expression of inflammation-associated genes in circulating leukocytes collected from horses with gastrointestinal tract disease. Am J Vet Res.

[13] Nieto JE, MacDonald MH, Braim AE, Aleman M (2009). Effect of lipopolysaccharide infusion on gene expression of inflammatory cytokines in normal horses in vivo. Equine Vet J.

[14] Fossum C, Hjertner B, Olofsson KM, Lindberg R, Ahooghalandari P, Camargo MM (2012). Expression of tlr4, md2 and cd14 in equine blood leukocytes during endotoxin infusion and in intestinal tissues from healthy horses. Vet Immunol Immunopathol.

[15] Tadros EM, Frank N (2012). Effects of continuous or intermittent lipopolysaccharide administration for 48 hours on the systemic inflammatory response in horses. Am J Vet Res.

[16] Henneke DR, Potter GD, Kreider JL, Yeates BF (1983). Relationship between condition score, physical measurements and body fat percentage in mares. Equine Vet J.

[17] Aiello SEE (1998). The Merck veterinary manual.

[18] Schroeder A, Mueller O, Stocker S, Salowsky R, Leiber M, Gassmann M (2006). The RIN: an RNA integrity number for assigning integrity values to RNA measurements. BMC Mol Biol.

[19] Skovgaard K, Cirera S, Vasby D, Podolska A, Breum SO, Durrwald R (2013). Expression of innate immune genes, proteins and microRNAs in lung tissue of pigs infected experimentally with influenza virus (H1N2). Innate Immun.

[20] Andersen CL, Jensen JL, Orntoft TF (2004). Normalization of real-time quantitative reverse transcription-PCR data: a model-based variance estimation approach to identify genes suited for normalization, applied to bladder and colon cancer data sets. Cancer Res.

[21] Vandesompele J, De Preter K, Pattyn F, Poppe B, Van Roy N, De Paepe A, et al. Accurate normalization of real-time quantitative RT-PCR data by geometric averaging of multiple internal control genes. Genome biology. 2002, 3(7). RESEARCH0034. 10.1186/gb-2002-3-7-research0034 PMC126239 12184808

[22] Moore JN, Norton N, Barton MH, Hurley DJ, Reber AJ, Donovan DC (2007). Rapid infusion of a phospholipid emulsion attenuates the effects of endotoxaemia in horses. Equine Vet J.

[23] Morris DD, Moore JN, Crowe N, Moldawer LL (1992). Effect of experimentally induced endotoxemia on serum interleukin-6 activity in horses. Am J Vet Res.

[24] Wearn JG, Suagee JK, Crisman MV, Corl BA, Hulver MW, Hodgson DR (2012). Effects of the insulin sensitizing drug, pioglitazone, and lipopolysaccharide administration on markers of systemic inflammation and clinical parameters in horses. Vet Immunol Immunopathol.

[25] Baskett A, Barton MH, Norton N, Anders B, Moore JN (1997). Effect of pentoxifylline, flunixin meglumine, and their combination on a model of endotoxemia in horses. Am J Vet Res.

[26] Jacobs CC, Holcombe SJ, Cook VL, Gandy JC, Hauptman JG, Sordillo LM (2013). Ethyl pyruvate diminishes the inflammatory response to lipopolysaccharide infusion in horses. Equine Vet J.

[27] Fessler JF, Bottoms GD, Coppoc GL, Gimarc S, Latshaw HS, Noble JK (1989). Plasma endotoxin concentrations in experimental and clinical equine subjects. Equine Vet J Suppl.

[28] Calvano SE, Xiao W, Richards DR, Felciano RM, Baker HV, Cho RJ (2005). A network-based analysis of systemic inflammation in humans. Nature.

[29] Talwar S, Munson PJ, Barb J, Fiuza C, Cintron AP, Logun C (2006). Gene expression profiles of peripheral blood leukocytes after endotoxin challenge in humans. Physiol Genomics.

[30] McLoughlin K, Turteltaub K, Bankaitis-Davis D, Gerren R, Siconolfi L, Storm K (2006). Limited dynamic range of immune response gene expression observed in healthy blood donors using RT-PCR. Mol Med.

[31] Wong HR (2012). Genetics and genomics in pediatric septic shock. Crit Care Med.

[32] Prabhakar U, Conway TM, Murdock P, Mooney JL, Clark S, Hedge P (2005). Correlation of protein and gene expression profiles of inflammatory proteins after endotoxin challenge in human subjects. DNA Cell Biol.

[33] de Sousa AR, Penalva LO, Marcotte EM, Vogel C (2009). Global signatures of protein and mRNA expression levels. Mol Biosyst.

[34] Vogel C, Marcotte EM (2012). Insights into the regulation of protein abundance from proteomic and transcriptomic analyses. Nat Rev Genet.

[35] Hollams EM, Giles KM, Thomson AM, Leedman PJ (2002). MRNA stability and the control of gene expression: implications for human disease. Neurochem Res.

[36] Tadros EM, Frank N, Donnell RL (2013). Effects of equine metabolic syndrome on inflammatory responses of horses to intravenous lipopolysaccharide infusion. Am J Vet Res.

[37] Fry DE (2012). Sepsis, systemic inflammatory response, and multiple organ dysfunction: the mystery continues. Am Surg.

[38] Gomez DE, Alonso DF, Yoshiji H, Thorgeirsson UP (1997). Tissue inhibitors of metalloproteinases: structure, regulation and biological functions. Eur J Cell Biol.

[39] Gaur U, Aggarwal BB (2003). Regulation of proliferation, survival and apoptosis by members of the TNF superfamily. Biochem Pharmacol.

[40] De Freitas I, Fernandez-Somoza M, Essenfeld-Sekler E, Cardier JE (2004). Serum levels of the apoptosis-associated molecules, tumor necrosis factor-alpha/tumor necrosis factor type-I receptor and Fas/FasL, in sepsis. Chest.

[41] Turrel-Davin F, Guignant C, Lepape A, Mougin B, Monneret G, Venet F (2010). Upregulation of the pro-apoptotic genes BID and FAS in septic shock patients. Crit Care.

[42] Weber SU, Schewe JC, Lehmann LE, Muller S, Book M, Klaschik S (2008). Induction of Bim and Bid gene expression during accelerated apoptosis in severe sepsis. Crit Care.

[43] Vallet B (2003). Bench-to-bedside review: endothelial cell dysfunction in severe sepsis: a role in organ dysfunction?. Crit Care.

[44] Satué K, Muñoz A, Gardón JC (2014). Interpretation of the equine leukogram. J Hematology Res.

[45] Whitney AR, Diehn M, Popper SJ, Alizadeh AA, Boldrick JC, Relman DA (2003). Individuality and variation in gene expression patterns in human blood. Proc Natl Acad Sci U S A.

[46] Eady JJ, Wortley GM, Wormstone YM, Hughes JC, Astley SB, Foxall RJ (2005). Variation in gene expression profiles of peripheral blood mononuclear cells from healthy volunteers. Physiol Genomics.

[47] Skibsted S, Bhasin MK, Aird WC, Shapiro NI (2013). Bench-to-bedside review: Future novel diagnostics for sepsis - a systems biology approach. Crit Care.

[48] Pierrakos C, Vincent JL (2010). Sepsis biomarkers: a review. Crit Care.

[49] Sutherland A, Thomas M, Brandon RA, Brandon RB, Lipman J, Tang B (2011). Development and validation of a novel molecular biomarker diagnostic test for the early detection of sepsis. Crit Care.

[50] Andaluz-Ojeda D, Bobillo F, Iglesias V, Almansa R, Rico L, Gandia F (2012). A combined score of pro- and anti-inflammatory interleukins improves mortality prediction in severe sepsis. Cytokine.

[51] Wong HR, Lindsell CJ, Pettila V, Meyer NJ, Thair SA, Karlsson S (2014). A multibiomarker-based outcome risk stratification model for adult septic shock. Crit Care Med.

[52] Declue AE, Johnson PJ, Day JL, Amorim JR, Honaker AR (2012). Pathogen associated molecular pattern motifs from Gram-positive and Gram-negative bacteria induce different inflammatory mediator profiles in equine blood. Vet J.

