# Supplementary material for: Dynamic expression of leukocyte innate immune genes in whole blood from horses with lipopolysaccharide-induced acute systemic inflammation
Source: BMC Vet Res. 2015 Jun 16;11:134. doi: 10.1186/s12917-015-0450-5 (PMC4467047; doi:10.1186/s12917-015-0450-5)
Supplement: Additional file 1: — Primers and reaction conditions. PCR efficiencies and correlation coefficients for genes marked with (*) are calculated based on a specific high-responder standard curve made from dilution series of a pool of cDNA samples showing high expression levels of the implicated genes. Genes marked with (#) were excluded from the study due to high sensitivity to genomic DNA contamination, indications of splice variants, or low primer efficiency. r2 = correlation coefficient. [file 12917_2015_450_MOESM1_ESM.pdf]

Gene functional class	Gene symbol	Gene name	Transcript ID	Sequence 5´-3´	Amplicon length (bp)	PCR efficiency	r^2
Interleukins	IL1B	Interleukin 1-beta	ENSECAT00000000066	F: CCTACAGCTGGAGACAGTAGACC<br>R: TGGGGTACATTGCAGACTCA	119	1.03	0.99
	IL2	Interleukin 2 (*)	ENSECAT00000016535	F: TCTGGGACTAAAGGGGTCTG<br>R: TGCTTTGACAAAAGGTAATCCA	104	0.93	0.98
	IL4	Interleukin 4	ENSECAT00000008569	F: CTGCAAAGGTGCTTCAACAG<br>R: TTGAGGTTCCTGTCCAGTCC	85	0.95	0.99
	IL6	Interleukin 6 (*)	ENSECAT00000017492	F: GCAAAAGATGAAGAATCCAGAAG<br>R: TCTGTGAATGCAGCTTAGCC	80	0.90	0.98
	IL8	Interleukin 8	ENSECAT00000016212	F: CGTTTTTGAAGAGAGCTGAGG<br>R: GCTTGAAGTTTCATTGGCATC	114	1.05	0.99
	IL10	Interleukin 10	ENSECAT00000009338	F: GCCTTCAGTAAGCTCCAAGAG<br>R: CCCTAGGATGCTTCAGTTTTTC	117	1.08	0.97
	IL15	Interleukin 15	ENSECAT00000015025	F: GAGGCTGGCATTCATGTCTT<br>R: CTGCCAGTTTGCCTCTGTTT	72	1.02	0.99
	IL17	Interleukin 17	ENSECAT00000004849	F: GGAGGCATGAAAGGATCAAG<br>R: TCAGATGGGTAAACCACAAGAA	85	1.08	0.97
	IL18	Interleukin 18	ENSECAT00000016149	F: TGGCAGGCTTGAACCTAAAC<br>R:CACAGGTTGATTTCCCTGGT	82	1.03	1.00
	IL1RN	Interleukin 1 receptor antagonist	ENSECAT00000005750	F: ACAAATGTGGCTCCTCCAAG<br>R: TTTCAGAGCGTCAGAAGTGC	111	1.03	1.00
TNF super family	TNF	Tumor necrosis factor	ENSECAT00000002048	F: GTTGTAGCAAACCCCCAAG<br>R: GGTTGTCTGTCAGCTTCACG	94	1.11	0.99
Growth factors	TGFB1	Transforming growth factor-beta 1	ENSECAT00000012048	F: CTGTTTCAGCTCCACAGAGAAG<br>R: AGAAGTTGGCGTGGTAGCC	113	1.04	0.99
	CSF2	Granulocyte-macrophage colony stimulating factor (#)	ENSECAT00000009868	F: TTCCTGTGCAACCCAGATG<br>R: GGAAGGCCTGCCTTACTTCT	114	–	–
Pattern recognition receptors	TLR4	Toll-like receptor 4	ENSECAT00000010707	F: GCCAGGGAAAGTCAACTCAA<br>R: TGGGAGACGATGTCCTTTTC	94	1.03	0.99
	TLR9	Toll-like receptor 9	ENSECAT00000014884	F: TGGTAATCCTGAGCCCTGAC<br>R: GGGCCAGAAGAGGACACTC	86	0.94	0.98
	CD14	CD14	ENSECAT00000001658	F: CTGCCCGTGGTGAGTAATCT<br>R: GGAGTTCTGGTCTTGCTGCT	81	1.04	1.00
Lymphokines	MIF	Macrophage migration inhibitory factor	ENSECAT00000013321	F: CAAGCCAGCCCAGTACATC<br>R: CAATCTTCCCGATGCTGTG	110	0.99	0.99
Transcription factors	NKAP	NF-κB activating protein	ENSECAT00000022623	F: CTATGGCCATGCTCTGTTACC<br>R: GCCAATTTCACCTCTTCGTG	91	1.04	0.99
Damage-associated molecular patterns	HMGB1	High mobility group box 1	ENSECAT00000003508	F: CACTGCTGCAGATGACAAGC<br>R: GCAGCAGGTTTTCCTTTAGC	105	1.01	0.99
Adhesion molecules	ITGAM	CD11b, integrin alpha M	ENSECAT00000019583	F: GTGAGGGCTCAGACGGATAC<br>R: CCAGTGATGAGAGCCAGGAG	110	1.01	0.99
	ITGAX	CD11c, integrin alpha X	ENSECAT00000026050	F: AGAGCCCAGATGGAGATGG<br>R: CACAGAGCTGCCCACAATAA	75	1.02	1.00
	SELL	Selectin L	ENSECAT00000021448	F: CAACCTGTCAAGTGATTCAGTG<br>R: AGCAGTTGAAGGTGCATGTG	114	0.99	0.99
Kinases	MAPK14	Mitogen-activated protein kinase 14, P38 mitogen-activated protein kinase	ENSECAT00000015606	F: GATGAATGGAAAAGCCTGACC<br>R: AGTGAAGCGGGATCAAGAGA	116	1.06	0.99

Gene functional class	Gene symbol	Gene name	Transcript ID	Sequence 5´-3´	Amplicon length (bp)	PCR efficiency	r^2
Apoptosis related molecules	FAS	FAS cell surface death receptor	ENSECAT00000014464	F: CCCAGGACTATTGTCTATTTTTCAG<br>R: CCATTCTTCCGAGCAAATTC	107	0.96	1.00
	BID	BH3 interacting domain death agonist	ENSECAT00000006654	F: GGAGGAAGACAGAAGACAGTGC<br>R: GAGCATGGACAACATCAGCA	106	1.03	1.00
	CASP3	Caspase 3	ENSECAT00000023864	F: CACAGCACCTGGTTACTATTCC<br>R: GCGTACAGCTTCAGCATAGC	87	1.05	1.00
	BCL2L1	BCL2-like 1, BCL-XL	ENSECAT00000018124	F: GGCTGGGACACCTTTGTG<br>R: AGAACCACACCAGCCACAGT	116	1.07	0.99
Enzymes	MMP8	Matrix metallopeptidase 8	ENSECAT00000022856	F: CTGGAGATATGATAACCAAAGACG<br>R: CTTGCTGGAAAACTGCATCA	107	1.11	1.00
	TIMP1	Metallopeptidase inhibitor 1	ENSECAT00000014937	F: GGGGAATGCTCAGTGTTTC<br>R: TCTGGAAGCCCTTGTCAGAG	110	1.03	0.99
	MPO	Myeloperoxidase (#)	ENSECAT00000007644	F: CGATGGTGATCGGTTTTG<br>R: GTGGTGATGCCTGTGTTGTC	114	0.90	–
Cytokine receptors	IL6ST	Interleukin 6 signal transducer	ENSECAT00000021352	F: GCCCAGACAACAGTATTTCAAAC<br>R: CTGATGAACCTTGCTTTGACC	92	1.03	0.99
Chemokines	CCL5	Chemokine (C-C motif) ligand 5	ENSECAT00000026840	F: CCAGCAGTCGTCTTTGTCAC<br>R: GCCCTCCAATCCTAGCTCAT	110	1.06	0.99
Reactive oxygen species	SOD2	Superoxid dismutase 2	ENSECAT00000012435	F: CTGCAGGGAACAACAGGTC<br>R: CACATTCCAAATGGCTTTCA	114	1.01	0.99
Housekeeping genes	TBP	TATA box binding protein	ENSECAT00000008864	F: CACCAGCAGTTTAGTAGTTATGAGC<br>R: AGGAGAACAATTCTGGGTTTGA	80	1.10	0.99
	DIMT1	Dimethyladenosine transferase 1 homolog	ENSECAT00000014730	F: GACTTCATCAGATTGCTACATGG<br>R: TTCCAGTTTCTTGACTTGAGTTTG	100	1.04	1.00
	SDHA	Succinate dehydrogenase complex, subunit A	ENSECAT00000003106	F: AAGACCGGGAAGGTCTCG<br>R: TCCGTTCCCATCAGTAGGAG	109	1.01	1.00
	ACTB	Actin-beta	ENSECAT00000016856	F: CAGTGGCATCCACGAAACTA<br>R: AGCACTGTGTTGGCGTACAG	84	1.02	0.99
	HPRT1	Hypoxanthine phosphoribosyltransferase 1	ENSECAT00000018496	F: CAGGACTGAACGGCTTGC<br>R: CCAGCAGGTCAGCAAAGAAT	107	1.01	0.99
	B2M	Beta-2-microglobulin	ENSECAT00000001009	F: TTACTCACGTCACCCAGCAG<br>R: ATTTCAATCTCAGGCGGATG	84	1.12	0.974
	GAPDH	Glyceraldehyd-3-phosphate dehydrogenase	ENSECAT00000023721	F: CAAGCTCATTTCCTGGTATGAC<br>R: TTACTCCTTGGAGGCCATGT	85	1.09	0.994
