# Supplementary figures and images for: Dynamic expression of leukocyte innate immune genes in whole blood from horses with lipopolysaccharide-induced acute systemic inflammation
Source: BMC Vet Res. 2015 Jun 16;11:134. doi: 10.1186/s12917-015-0450-5 (PMC4467047; doi:10.1186/s12917-015-0450-5)

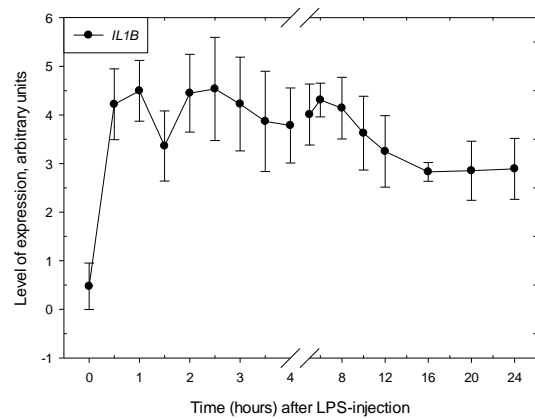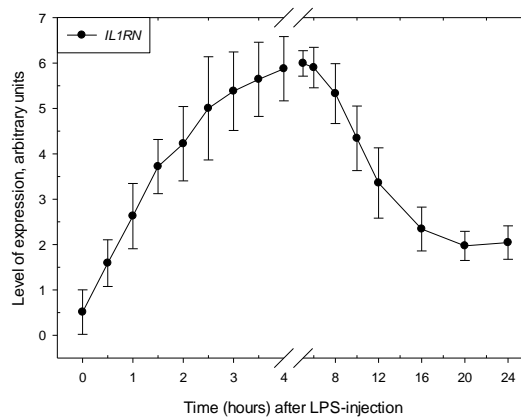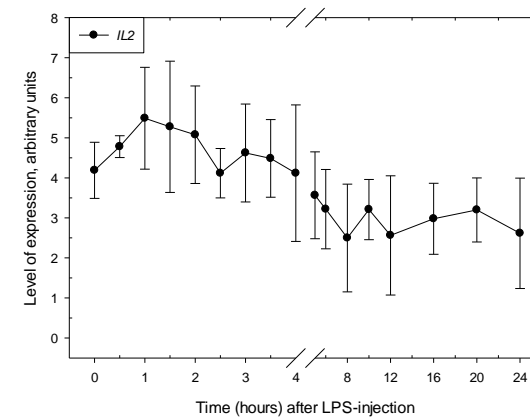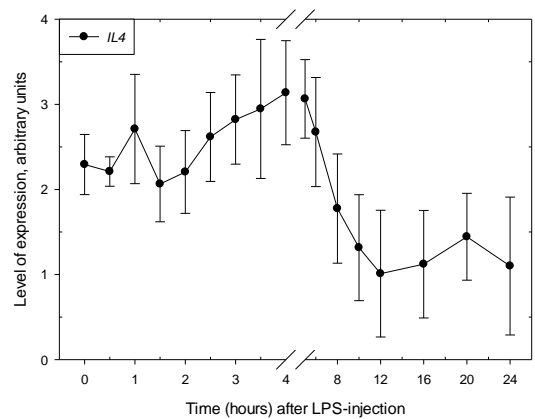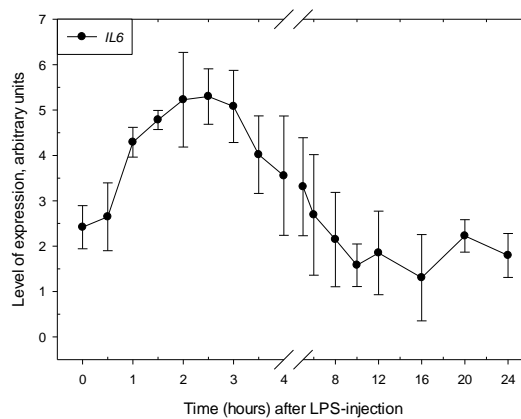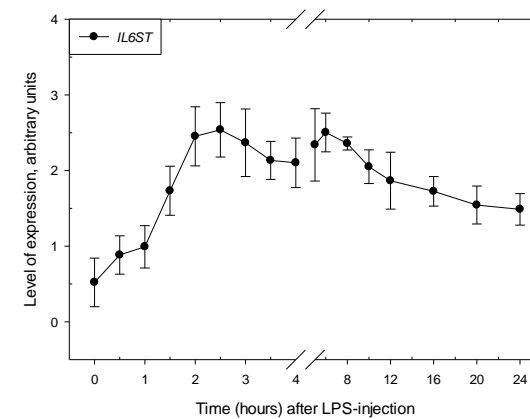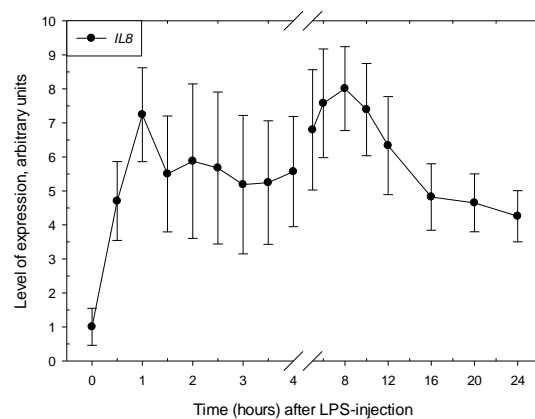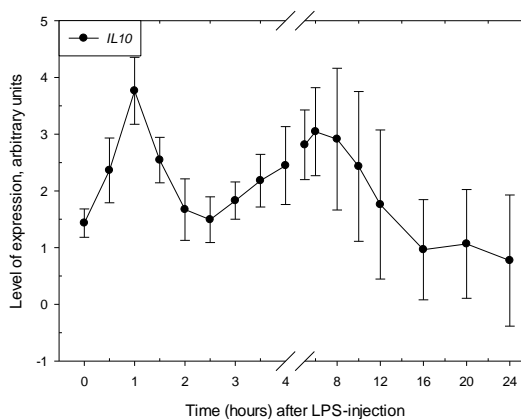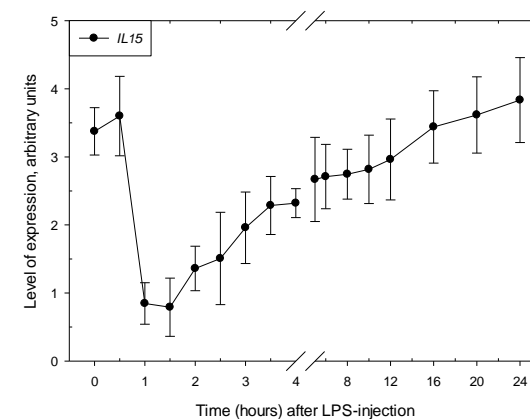

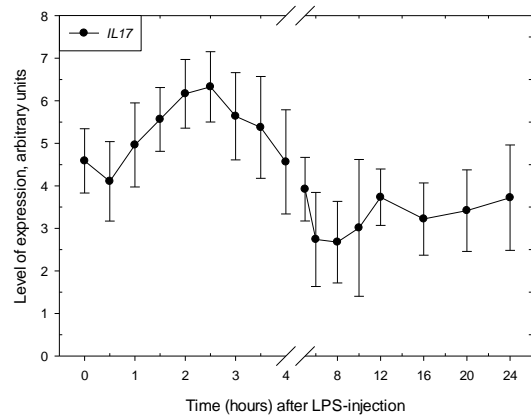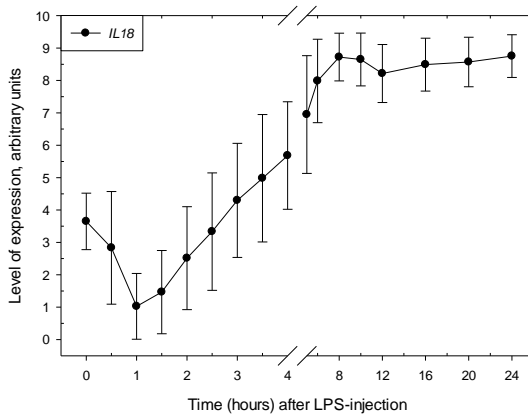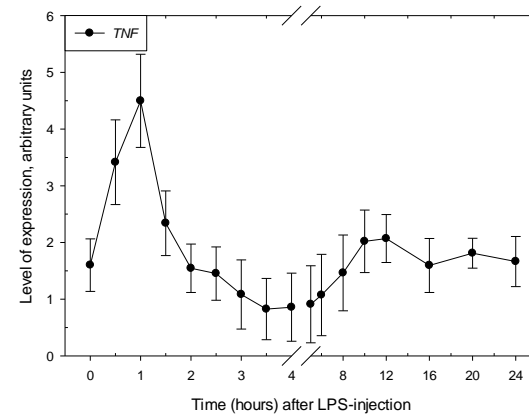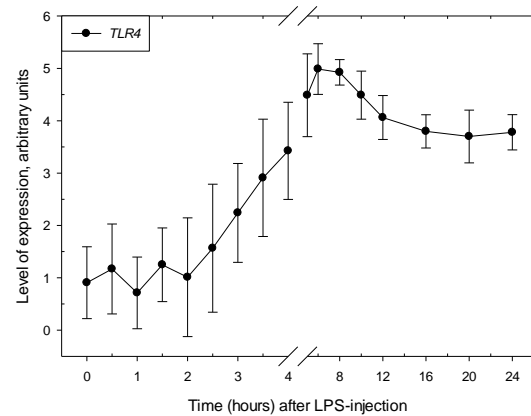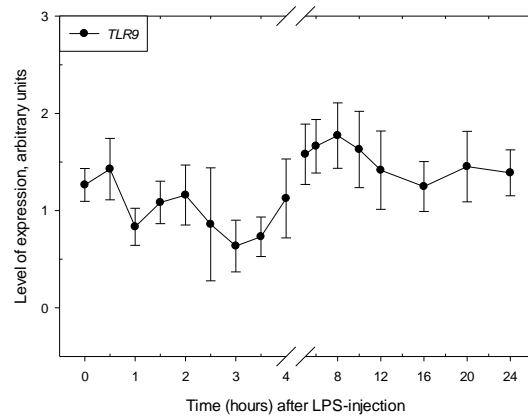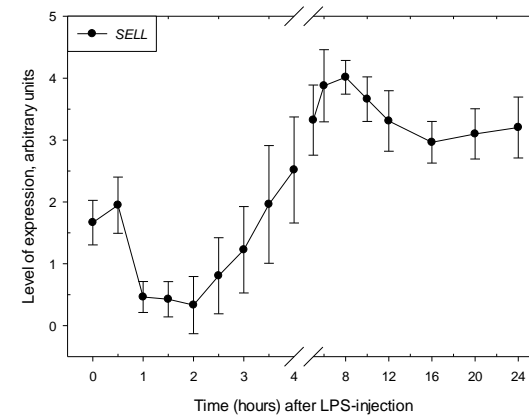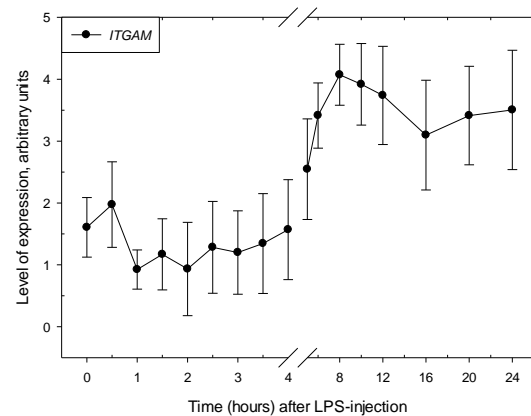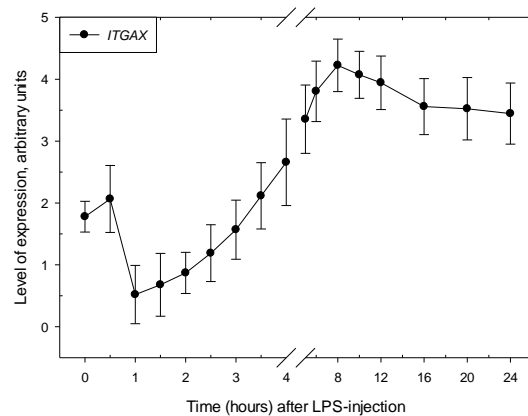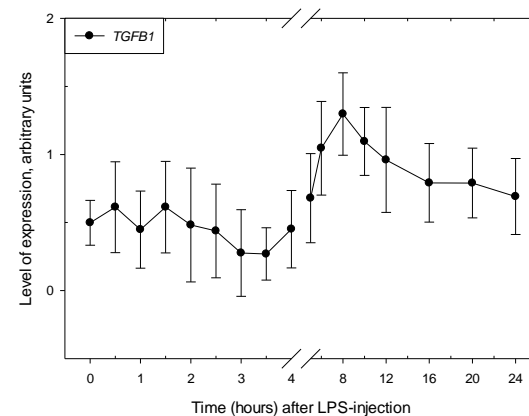

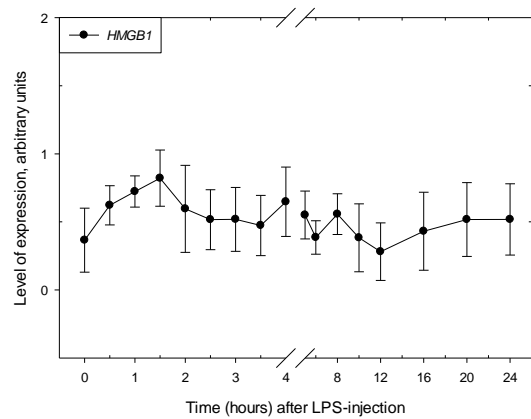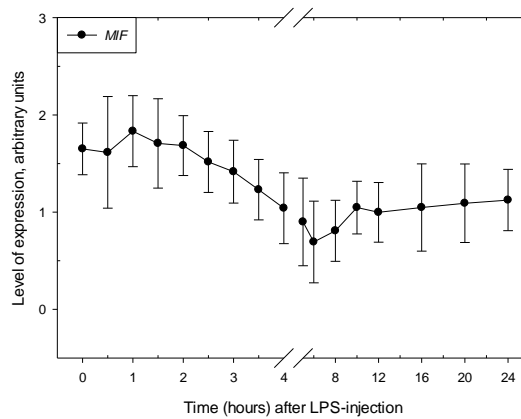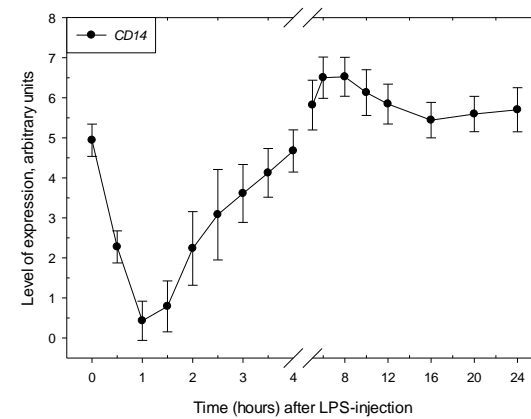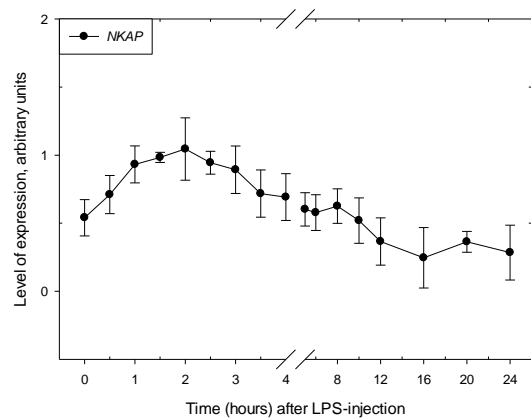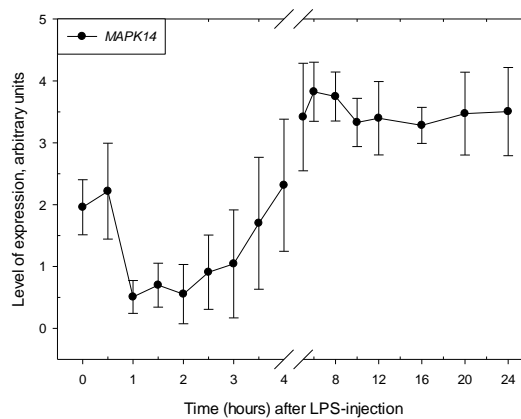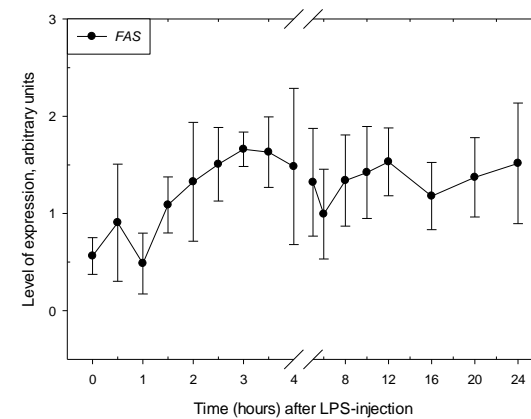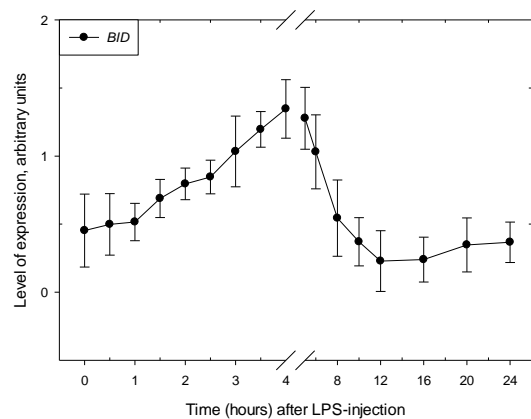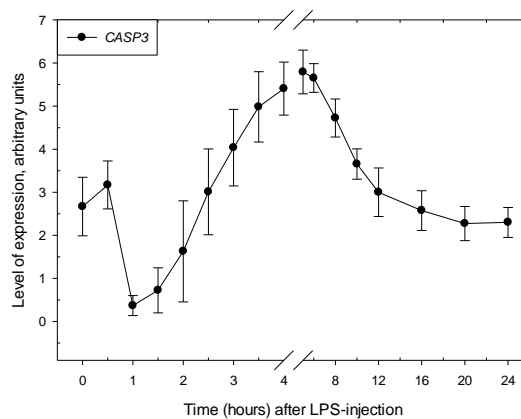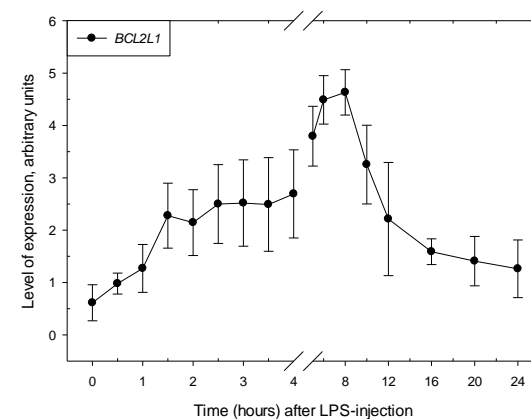

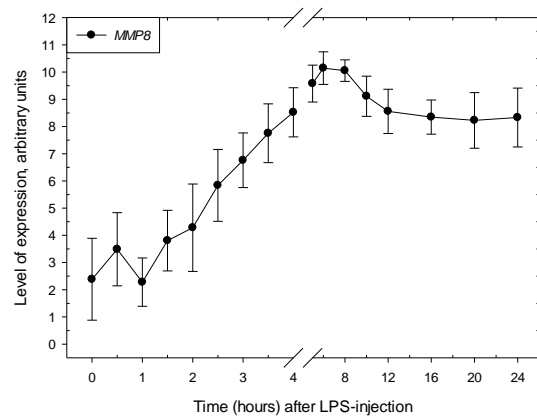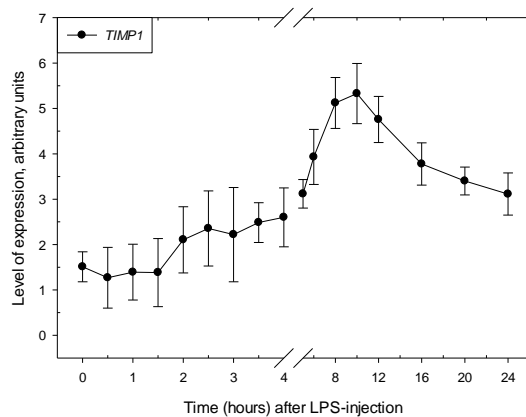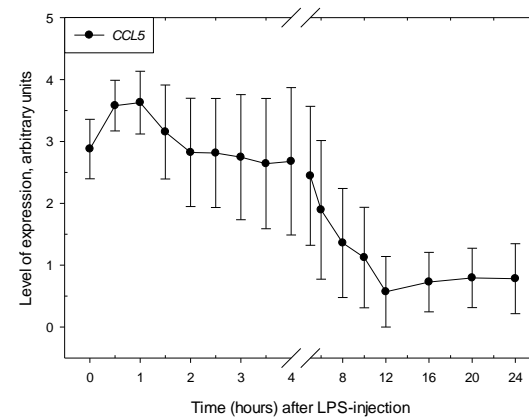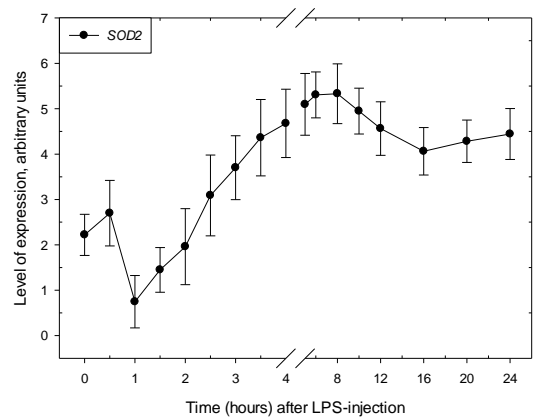

Supplement: Additional file 2: — Post-induction mean expression levels for each gene. Log2-transformed relative gene expressions (mean ± SD) depicted as a function of time in hours after LPS-injection. Expression levels are set relative to the lowest expression level of the gene in question. Time point 0 indicates baseline levels immediately before LPS-injection. Time after injection is depicted non-equidistantly. [file 12917_2015_450_MOESM2_ESM.pdf]
